# Primary headache in the elderly in South-East Asia

**DOI:** 10.1007/s10194-012-0434-9

**Published:** 2012-03-16

**Authors:** Mei-Ling Sharon Tai, Jaishree Sharmini Jivanadham, Chong Tin Tan, Vijay K. Sharma

**Affiliations:** 1 Division of Neurology, Department of Medicine, University of Malaya, 50603 Kuala Lumpur, Malaysia; 2 Division of Neurology, National University Health System, Singapore, Singapore

**Keywords:** Headache, Elderly, Young, Clinic, Ethnic

## Abstract

Headache aetiology and presentation are considerably different in elderly individuals. However, literature on headache characteristics among Asians is limited. The objective of this study was to evaluate the headache characteristics among elderly in an outpatient clinic setting in Malaysia, a South-East Asian country with diverse ethnicity. In this prospective cross-sectional study, patients presenting with headache to Neurology and Primary Care Clinics of University Malaya Medical Centre between February 2010 and July 2010 were included. Data for consecutive eligible adult patients were entered in a prospective headache registry. International Headache Criteria II (ICHD-II) were used to classify various headache subtypes. Patients with headache due to intracranial space occupying lesions were excluded. Patient were divided into two age groups—elderly (55 years and above) and younger (less than 55 years of age). Of the 175 screened patients, 165 were included in the study—70 in elderly age group and 95 in younger group. Tension-type headache was the commonest subtype (45.7 %) among the elderly while Migraine without aura (54.7 %) was more common in young adults. More elderly patients suffered from chronic daily headache as compared to younger patients (47.1 vs. 28.4 %; *p* = 0.015). Headache subtypes and frequency differ considerably among elderly South East Asian patients.

## Introduction

Headache is one of the most common conditions seen in general medicine as well as neurology specialist clinics [1]. Prevalence of headache in general population during 1 year has been reported as about 46 % with the life-time prevalence of 64 % [2]. Headache among the elderly has been evaluated only by a few studies [1, 3–7]. Although, the prevalence of headache decreases with age [3, 8], it still remains a common complaint in the elderly [8, 9]. Most of the previous studies on headache in the elderly have included Caucasian patients. Compared to males, females tend to have more headaches in all age groups [8–10]. Accordingly, among the elderly aged 75 years or more, the prevalence of headache was 55 % in females compared to 22 % males [8, 9]. Expectedly, higher prevalence rates were reported among younger population with female preponderance (66 % females and 55 % males in patients aged 55–74 years; 92 % females and 74 % males in younger adults aged 21–34 years) [8–10]. Although, headache starts at younger ages, de novo appearance in elderly may be observed in 5.4 % patients aged 65 years old and above [6]. Furthermore, all subtypes of headache are observed among elderly. However, migraine occurs less frequently (4.6 %) as compared to tension-type headache (16–27 %) [5, 8].

Various demographic and social factors influence the prevalence of headache. Accordingly, the prevalence of headache in rural Ecuador was found to be considerably lower (6.8 % among elderly) as compared to the Western literature [11]. Data for headache among the elderly in Asia are scarce. In a study conducted in a nursing home in Thailand, 1-year headache prevalence of 55 % was similar to Western studies [1]. However, a community study on Chinese elderly patients in Taiwan reported a relatively lower prevalence (38 %) of headache, with 1-year prevalence of tension-type headache in 20 % of men and 46 % of women [12]. Migraine was much less common and occurred in only 0.7 % in men and 4.7 % in women in this study [12]. Similar patterns of lower prevalence of migraine among the elderly were observed in other community studies (0.27–0.76 % and 0.3 % in China and Hong Kong, respectively) [13–15].

While it is true that community-based study on headache is essential, it is equally important to study the characteristics of headache presenting to the clinic. This is because these are the headaches that burden the patient, which may be different from those ascertained by community based surveys. For example, a study from Denmark showed that 16 % of migraine patients as compared to only 4 % tension-type headache sought treatment from one or more specialists [16].

Malaysia is a developing country located in South East Asia. Its diverse ethnic distribution (consisting mainly of Malays, Chinese and Indians) may be considered a representative sample of Asian populations. There has been no previous clinic based study on frequency, characteristics and classification of headache among the elderly from Asia. We aimed to evaluate the characteristics of headache among the elderly in our Malaysian patients attending the outpatient clinics affiliated to our tertiary care center. We looked at the differences in the treatment seeking behavior for headache in our study cohort.

## Methods

### Patient selection

This was a prospective cross-sectional study done at the University Malaya Medical Centre between February 2010 and July 2010. The study was approved by the Institutional Ethics Committee of University Malaya Medical Centre. Consecutive patients, aged 18 years and more were recruited from the affiliated primary care and specialist neurology outpatient clinics. Patients referred to the neurology clinic and primary care clinic for headache were included in the study. All newcomers were also interviewed for the presence of headache. We evaluated the 3-month prevalence of headache in our study population. Patients with headache secondary to intracranial mass lesions were excluded. Informed consent was obtained from all the study participants or their legally acceptable representatives. Patients were divided into two groups: aged 18–54 years (called younger patients) and those aged 55 years and above (called elderly). The age of 55 years chosen as cut-off to identify elderly was based on the mandatory retirement age in Malaysia of 55 years [17]. Furthermore, selecting a higher cut-off age would have adversely affected our recruitment rates due to lower prevalence of headache in patients more than 55–60 years old [7].

### Study design

The study involved use of a structured headache questionnaire. The International Headache Society (IHS) classification, International Headache Criteria II (ICHD-II) were used to classify the headache subtypes [18]. Neuroimaging studies of the brain and/or cervical spine were performed, when necessary. All the patients were followed up in the neurology clinic after 1–5 months. Eleven patients unable to come to the clinic, due to various reasons, were interviewed through telephone.

### Data collection

We collected information about the demographic characteristics, marital status, significant past medical history and various questions related to the headache profile. Specific data collected about headache included the time of onset, frequency, site, character and intensity. We defined chronic daily headache (CDH) as a headache frequency more than 15 days in a month, with duration of more than 4 h a day [19]. The diagnosis of CDH and its subtypes was made on the initial interview. Use of various medications for pain relief was also assessed. We collected information about various non-pharmacological measures adopted by the patients during headache that included avoidance of certain food items that precipitated headache, use of wet towel or cold pack over head, avoiding the sun, lying quiet, tying a tight cloth around the forehead, traditional head and neck massage and local application of use of medicated oil. Data on trigger factors of headache such as stress, sun exposure, sleep deprivation, oversleep, missing meal and weather were also collected.

### Case definition

The diagnosis of various subtypes of headache was based on the International Headache Society (IHS) Criteria (ICHD II) [18]. Briefly, the more common types of headache were: migraine with aura (Typical aura with migraine headache), migraine without aura, chronic migraine, infrequent episodic tension-type headache, frequent episodic tension-type headache, chronic tension-type headache, cluster headache, trigeminal neuralgia and unspecified headache.

### Statistical analysis

All descriptive statistics were done using Statistical Package for Social Sciences, SPSS (Version 12.0, SPSS Inc., Chicago, USA). For categorical data, Chi-square test or Fisher’s test was used. Continuous variables were expressed as means and analyzed with Student’s *t* test. A *p* value of <0.05 was taken as statistically significant.

## Results

Out of the total of 1,020 patients who attended the participating outpatient clinics during the project, 175 (17.2 %) met our inclusion criteria of having history of headache at least once per month for more than 3 months. Finally, 168 patients were found to be eligible for the study; 90 % of the patients were recruited from neurology clinic and 10 % of the patients were from primary care clinic. The main reasons for exclusion were age less than 18 years old (5 cases) and intracranial mass lesion (2 cases). Three patients withdrew consent, leaving 165 cases for final analysis.

### Demographic characteristics

Of the 165 patients included in the final analyses, 70 (42.4 %) were elderly (mean age 64.6 ± 7.6 years) and 95 (57.6 %) were younger adults (mean age 29.8 ± 6.4 years). For the elderly patients, 32 patients were aged 55–64 years (45.7 % of all elderly patients), 32 in age group 65−74 years (45.7 % of all the elderly patients) and six aged 75–84 years (8.6 % of total elderly patients). Table 1 lists the demographic characteristics of the study population. Briefly, there were more female patients compared to male patients in both age groups. There was a fair representation of all major ethnicities in both age groups.

**Table 1:** Demographic characteristics of study population (*n* = 165)

Variable	Young (*n* = 95)	Elderly (*n* = 70)	*p* value
Age (mean ± SD)	29.8 ± 6.4	64.6 ± 7.6	<0.0001
Gender (*n*, %)			
Male	27 (28.4)	24 (34.3)	0.5
Female	68 (71.6)	46 (65.7)	
Ethnic groups (*n*, %)			
Malay	54 (56.8)	16 (22.9)	<0.0001
Chinese	12 (12.6)	27 (38.6)	<0.0001
Indian	25 (26.3)	26 (37.1)	0.17
Others	4 (4.3)	1 (1.4)	
BMI (kg/m^2^) (mean ± SD)	25.24 ± 5.92	25.92 ± 4.78	0.44
Marital status (*n*, %)			
Single	48 (50.5)	7 (10.0)	<0.0001
Married	42 (44.2)	46 (65.7)	0.007
Widow/widower	0	15 (21.4)	<0.0001
Divorced/separated	5 (5.3)	2 (2.9)	0.7

In the elderly, two patients had onset of migraine without aura, whereas one patient had onset of migraine with aura at ≥55 years old. Fifteen patients had onset of tension-type headache, two had trigeminal neuralgia and ten had unspecified headache at 55 years old and above. The age of onset for cluster headache was 61 years old whereas for glossopharyngeal neuralgia, onset of headache was 67 years old. Overall, 46 % of the elderly patients had onset of headache at ≥55 years old and headache starting at that age represented 19 % of headache in all ages.

In the young adults, the age of onset for migraine without aura was 10–39 years old, migraine with aura 11–34 years old, tension-type headache 10–38 years old, cluster headache 30–35 years old, trigeminal neuralgia 37 years old and unspecified headache 10–30 years old.

### Headache characteristics

Headache characteristics in the two age groups in our study population are represented in Table 2. Briefly, more elderly patients suffered from headaches everyday compared to the young (41.4 vs. 14.7 %; *p* < 0.0001). Headache on more than 15 days in a month occurred more frequently among the elderly as compared to the younger patients (47.1 vs. 28.4 %; *p* = 0.015). Headache of recent onset was noted in three elderly (two with unspecified headache and one with episodic tension-type headache) and one younger patient (with episodic tension-type headache).

**Table 2:** Comparison of headache characteristics among young and elderly

Variable	Young (*n* = 95)	Elderly (*n* = 70)	*p* value
Time of onset (*n*, %)			
On waking up	14 (14.7)	25 (35.7)	0.003
Afternoon/evening	21 (22.1)	15 ((21.4)	1.0
Night	7 (7.4)	2 (2.9)	0.3
Anytime	53 (55.8)	28 (40)	0.06
Frequency (*n*, %)			
Everyday	14 (14.7)	29 (41.4)	<0.0001
Every other day	10 (10.5)	7 (10)	1.0
>1–3×/week	40 (42.1)	22 (31.4)	0.19
1×/week–1×/2 week	15 (15.8)	3 (4.3)	0.02
<1×/2 week–1×/month	7 (7.4)	5 (7.1)	1.0
<1×/month–1×/6 months	7 (7.4)	1 (1.4)	0.14
<1×/6 months–1×/year	2 (2.1)	3 (4.3)	0.65
Number of headache in a month (*n*, %)			
Headache ≤15 days/month	68 (71.6)	37 (52.9)	0.015
Headache >15 days/month	27 (28.4)	33 (47.1)	0.015
Site of pain (*n*, %)			
Frontal	12 (12.6)	9 (12.9)	1.0
Temporal	47 (49.5)	18 (25.7)	0.002
Occipital	16 (16.8)	20 (28.6)	0.09
Vertex	11 (11.6)	7 (10)	0.81
Whole head	6 (6.3)	5 (7.1)	1.0
Migrating	1 (1.1)	1 (1.4)	1.0
Face	1 (1.1)	7 (10)	0.01
Back of neck	1 (1.1)	3 (4.3)	0.31
Character of headache (*n*, %)			
Throbbing/pulsating	61 (64.2)	32 (45.7)	0.02
Sharp/stabbing	19 (20.0)	12 (17.1)	0.69
Tightness/pressing	14 (14.7)	18 (25.7)	0.11
Not specific	1 (1.1)	8 (11.4)	0.005
Intensity of headache (*n*, %)			
Mild	9 (9.5)	28 (40)	<0.0001
Moderate	45 (47.4)	19 (27.1)	0.01
Severe	41 (43.2)	23 (32.9)	0.19

Temporal headaches were seen more commonly among the younger patients (49.5 vs. 25.7 % in the elderly; *p* = 0.002), largely due to the higher prevalence of migraine. Similarly, facial pain was more common among the elderly (10 vs. 1.1 % in younger age; *p* = 0.01) due to higher prevalence of trigeminal neuralgia.

### Types of headache according to ICHD II classification

Headache subtypes differed in the two age groups (Table 3). While migraine without aura (54.7 %) and migraine with aura (16.8 %) were more common among the younger patients, the commonest headache in the elderly was tension-type headache (45.7 %) and half of them were chronic in nature. Twelve young adults and four elderly patients suffered from chronic migraine. None of the patients fulfilled the diagnostic criteria of medication-overuse headache.

**Table 3:** Headache classification according to age group

Headache classification	Young (*n* = 95, %)	Elderly (*n* = 70, %)	*p* value
Migraine without aura	52 (54.7)	9 (12.9)	<0.0001
Migraine with aura	16 (16.8)	4 (5.7)	0.032
Chronic migraine	12 (12.6)	4 (5.7)	0.19
Infrequent and frequent episodic tension-type headache	11 (11.6)	16 (22.9)	0.06
Chronic tension-type headache	5 (5.3)	16 (22.9)	0.002
Cluster headache	2 (2.1)	1 (1.4)	1.0
Trigeminal neuralgia	1 (1.1)	5 (7.1)	0.08
Glossopharyngeal neuralgia	0 (0)	1 (1.4)	0.424
Unspecified headache	8 (8.4)	18 (25.7)	0.004

As for CDH, five out of 16 elderly patients with chronic tension-type headache had the onset aged less than 55 years old. Headache onset in three out of four elderly patients with chronic migraine occurred before 55 years of age.

### Trigger factors for the headache

Sun exposure was the most common trigger in both age groups, followed by sleep deprivation, weather, missing meal and stress.

### Treatment seeking behavior for the headache

We did not observe any significant difference in the treatment seeking behavior for headache in the two age groups. Overall, avoidance of food items that precipitated headache such as chocolates and coffee was widely practiced by both age groups and head-massaging was the commonest method used to relieve the headache.

Avoidance of food which precipitated headache was practiced by more migraine patients in the elderly and young adults. Five elderly patients who had migraine without aura, two patients with migraine with aura, two with tension-type headache, four with unspecified headache, one with glossopharyngeal neuralgia and one with trigeminal neuralgia avoided food which precipitated headache. In the younger group, avoidance of food items that precipitated headache was practiced by six patients with migraine with aura, 13 with migraine without aura, two with tension-type headache, two with unspecified headache and one patient with trigeminal neuralgia.

Head-massaging was practiced by more elderly and younger patients with tension-type headache. Head-massaging was used by nine patients with tension-type headache and two with unspecified headache in the elderly group. In the younger group, massaging was used by six patients with tension-type headache, five with migraine without aura, two with migraine with aura and two with unspecified headache.

## Discussion

Our study describes headache characteristics, subtypes and frequency among the young and elderly patients attending outpatient primary care and specialist clinic affiliated to our tertiary care center in Malaysia. Some of the headache features showed significant differences between the young and the elderly age groups. Despite significant differences in headache characteristics and subtypes, the treatment seeking behavior of the elderly patients were similar to the younger adults. Malaysia is a developing nation. Owing to a good mixture of the three major ethnicities (Chinese, Indians and Malays) in the region, our study population might be considered a good representative of South-East Asia.

Headache is a common disease among Malaysians with 1 year prevalence of 26.5 % for tension-type headache, 9.0 % for migraine and 28.2 % for other subtypes of headache [20]. In our study, 42.4 % patients were elderly (mean age 64.6 ± 7.6 years). Our findings are consistent with the previous community-based study in Malaysia by Alders et al. [20] that reported the prevalence of headache in various age groups; 46 % in patients before 15 years of age, 80 % in age group 16–25 years, 78 % in age group 26–35 years, 73 % in age group 36–45 years, 54 % in age group 46–55 years and 39 % in those more than 65 years old.

A significant proportion of elderly patients in our study suffered from chronic daily headaches (47.1 %) while the headache frequency was lower (less than 15 days/month) among the younger (71.6 %) patients. Perhaps, this might be explained by the relatively greater frequency of chronic tension-type headache in the elderly as compared to the migraine headache in the young. Our observations are consistent with the study by Langemark et al. [21] in which chronic headaches were frequent among the elderly, affecting about 10 % of women and 5 % of men above 70 years. Similar findings have been reported among the elderly in various community studies from Italy, Taiwan and Georgia [4, 22, 23].

A sizeable minority of the elderly patients that had CDH had such headaches starting at a younger age. This feature has been reported consistently in various population studies [23, 24]. Furthermore, these studies have reported the lack of decline in CDH prevalence. Interestingly, a recent large population-based study reported that despite the decline in the prevalence of episodic headache, the remission rates of chronic migraine are not lower in the elderly as compared to a younger age [24].

An important finding of our study is the considerably higher headache frequency among our elderly study participants. Most (94.3 %) of the Malaysian elderly headache patients reported at least one episode in a month, which is relatively much higher than the previously reported figures of 18.0 % in Italians [4] and 33 % among the elderly nursing home residents in Thailand (33 %) [1]. Although, difficult to substantiate, higher education level, cultural differences and physical independency could have contributed to the observed differences. The elderly patients with episodic headaches had an established pattern for a long time and did not seek care for headache. In comparison, the episodic headaches in the younger group were more likely to be of recent onset.

Another interesting feature of headache patients is the treatment seeking behavior in various age groups. While the elderly seek medical attention for tension-type headache for reassurance [25], younger patients are usually driven to the outpatient clinics due to the headache severity [4, 6, 21]. Although, we observed a similar pattern, this phenomenon occurred even when a larger proportion of study patients (60 %) presented with moderate-to-severe headache, which is comparatively higher than the prevalence of 20.4 % in an Italian study by Prencipe et al. [4]. Perhaps, the observed difference is due to the largely rural population in the Italian study [4]. One distinctive observation in our study is the higher proportion of chronic tension-type headache among the patients with tension headache. This is in contrast to the Bruneck Study done in Italy that reported a lower (35.8 %) 1 year prevalence of tension-type headache with only 5 % patients having chronic tension-type headache [26]. This could have occurred due to the hospital based nature of our study as compared to the population-based Bruneck study [26]. Our study was conducted in a tertiary hospital located in the urban Malaysia with easy and relatively cheaper medical care and transport services and these logistic factors might have accounted for the higher number of headache patients seeking medical treatment [25].

The influence of culture on the treatment seeking behavior was evident in our study. Accordingly, the practice of head and neck massage and the use of medicated oil, a common tradition of South East Asia [27, 28] were widely practiced by our study participants. Interestingly, the treatment seeking behavior did not differ between the young and the elderly patients, probably reflecting the underlying concepts, cultural factors and traditional attitudes to the illness. Head-massaging was used more by our patients with tension-type headache.

Zanchin et al. [29] reported that massage on the temples and nape was the most common maneuver used by tension-type headache patients.

Our study has certain limitations. First, our study might be considered to be small in size. Second, our study sample included largely an urban population attending the clinics affiliated to a tertiary center. The patients who attended the neurology clinic were referred patients. However, we believe that inclusion of consecutive and urban patients could have compensated for the small numbers by improving the quality of data.

In conclusions, we present the headache characteristics, subtypes and treatment seeking behavior among the multi-ethnic South-East Asian patients. Our findings would contribute to the limited information available on headache among Asians in general and among the elderly in particular. Perhaps, a larger Asia-wide study on headache is warranted to understand the contributions of ethnic/racial factors in the field of neurology of headache.

## References

[1] Srikiatkhachorn A (1991). Epidemiology of headache in the Thai elderly: a study in the Bangkae Home for the aged. Headache.

[2] Manzoni GC, Stovner LJ (2010). Epidemiology of headache. Handbook of Clinical Neurology.

[3] Ward TN (2002). Headache disorders in the elderly. Curr Treat Options Neurol.

[4] Prencipe M, Casini AR, Ferretti C, Santini M, Pezzella F, Scaldaferri N (2001). Prevalence of headache in an elderly population: attack frequency, disability, and use of medication. J Neurol Neurosurg Psychiatry.

[5] Camarda R, Monastero R (2003). Prevalence of primary headache in Italian elderly: prelimanary data from the Zabut aging project. Neurol Sci.

[6] Pascual J, Berciano J (1994). Experience in the diagnosis of headaches that start in the elderly. J Neurol Neurosurg Psychiatry.

[7] Lisotto C, Mainardi F, Maggioni F, Dainese F, Zanchin G (2004). Headache in the elderly: a clinical study. J Headache Pain.

[8] Capobianco DJ (2003) Headache in the elderly. Advanced studies in medicine 3(6C):S556–561

[9] Mariecken Fowler V, David Capobianco J, David Dodick W (2004). Headache in the Elderly. Sem Pain Med.

[10] Waters WE (1974). The Pontypridd headache survey. Headache.

[11] Sachs H, Sevilla F, Barberis P, Bolis L, Schoenberg B, Cruz M (1985). Headache in the rural village of Quiroga, Ecuador. Headache.

[12] Wang SJ, Liu HC, Fuh JL, Liu CY, Lin KP, Chen HM (1997). Prevalence of headaches in a Chinese elderly population in Kinmen: age and gender effect and cross-cultural comparisons. Neurology.

[13] Cheng XM, Cai K, Li SC, Wong WJ, Wu SP (1990). An epidemiologic survey of migraine in six cities in *China*. Chin J Neurol.

[14] Zhao F, Tsay JY, Cheng XM, Wong WJ, Li SC, Yao SX (1988). Epidemiology of migraine: a survey in 21 provinces of the People’s Republic of China, 1985. Headache.

[15] Wong TW, Wong KS, Yu TS, Kay R (1995). Prevalence of migraine and other headaches in Hong Kong. Neuroepidemiology.

[16] Rasmussen BK, Jensen R, Olesen J (1992). Impact of headache on sickness absence and utilisation of medical services: a Danish population study. J Epidemiol Community Health.

[17] Masud J, Haron SA, Gikonyo LW (2008). Gender differences in income sources of the elderly in Peninsular Malaysia. J Fam Econ.

[18] ICHD-II International Headache Society Classification. http://ihs-classification.org/en/

[19] Lu SR, Fuh JL, Chen WT, Juang KD, Wang SJ (2001). Chronic daily headache in Taipei, Taiwan: prevalence, follow-up and outcome predictors. Cephalalgia.

[20] Alders EEA, Hentzen A, Tan CT (1996). A community-based prevalence study on headache in Malaysia. Headache.

[21] Langemark M, Olsen J, Poulsen DL, Bech P (1988). Clinical characterization of patients with chronic tension headaches. Headache.

[22] Katsarava Z, Dzagnidze A, Kukava M, Mirvelashvili E, Djibuti M, Janelidze M (2009). Primary headache disorders in the Republic of Georgia: prevalence and risk factors. Neurology.

[23] Wang SJ, Fuh JL, Lu SR, Liu CY, Hsu LC, Wang PN (2000). Chronic daily headache in Chinese elderly: prevalence, risk factors, and biannual follow-up. Neurology.

[24] Manack A, Buse DC, Serrano D, Turkel CC, Lipton RB (2011). Rates, predictors, and consequences of remission from chronic migraine to episodic migraine. Neurology.

[25] Wang SJ, Fuh JL, Young YH, Lu SR, Shia BC (2001). Frequency and predictors of Physician consultations for headache. Cephalalgia.

[26] Schwaiger J, Kiechl S, Seppi K, Sawires M, Stockner H, Erlacher T (2009). Prevalence of primary headaches and cranial neuralgias in men and women aged 55–94 years (Bruneck Study). Cephalalgia.

[27] Tan CT (1997). Cultural belief system and headache. Neurol J Southeast Asia.

[28] Buchwald D, Panwala S, Hooton TM (1992). Use of traditional health practices by Southeast Asian refugees in a primary care clinic. West J Med.

[29] Zanchin G, Maggioni F, Granella F, Rossi P, Falco L, Manzoni GC (2001). Self-administered pain-relieving manoeuvres in primary headaches. Cephalalgia.

